# Assessing the Feasibility of the Hybrid Ecological Therapeutic Intervention (HEI) for Preschoolers with ASD

**DOI:** 10.3390/children13010079

**Published:** 2026-01-04

**Authors:** Meir Lotan, Nophar Ben David, Merav Bibas

**Affiliations:** 1 Physical Therapy Department, Faculty of Health Sciences, Ariel University, 65th Ramat Hagolan St., Ariel 4070000, Israel; 2 TIPUL-LI AD HABAIT, 2nd Harokmim St., Holon 5885845, Israel; nopharbd@gmail.com (N.B.D.); merav@tipul-li.com (M.B.); 3 Carmel Hospital, 7th Michal St., Haifa 3103301, Israel; 4 Amichai, Non-Profit Organization for Individuals with Disabilities, 3rd Yakinton St., Hod HaSharon 4521643, Israel

**Keywords:** autism, Hybrid Ecological Intervention, pre-school children, ASRS, GAS, TOP-4, PLS-5

## Abstract

**Background**: Autism Spectrum Disorder (ASD) necessitates enhanced therapeutic support, especially in rural areas. Individual therapeutic sessions are costly, presenting an economic burden on the family of the child with ASD, as well as on healthcare and educational systems. Therefore, the current investigation aimed to assess the feasibility of a new hybrid therapeutic model involving a combination of remote and in situ interventions, ecologically implemented. **Methods**: The following outcome measures were used to assess the program’s feasibility and preliminary outcomes. The Preschool Language Scales 5th Edition (PLS-5), the Test of Playfulness 4th edition (TOP-4), and individually tailored goals evaluated using the Goal Attainment Scale (GAS) and the Autism Spectrum Rating Scale (ASRS). The evaluated children with ASD (N = 25), age range of 39–76 months (Mean: 53.1 ± 11.9), were treated with the novel Hybrid Ecological Intervention (HEI) method, where each child received bimonthly frontal therapeutic sessions and bi-weekly remote therapeutic sessions by a health care professional (OT or ST), supported by four weekly frontal sessions by a technological support person supervised by healthcare professionals. **Results**: All qualitative scales presented were associated with improvements in all evaluated areas. Qualitative data mostly supported the HEI and ways to overcome existing challenges, supporting the use of both evaluation methods. **Conclusions**: The use of quantitative and qualitative data was found to be efficient and complementary to one another. The scales used (ASRS, GAS) were found to be useful tools for this method and for these participants. The HEI model was found to be associated with improvement in play, communication, social abilities, as well as autism severity.

## 1. Introduction

Autism Spectrum Disorder (ASD) is an umbrella term for a wide range of behaviors associated with a neurodevelopmental disorder originating partly due to yet-unclear genetic components [1] along with various environmental influences in accordance with the specification set by the DSM-V [2]. It is a developmental disability detectable during childhood, with an individual clinical manifestation which can change during the lifespan of the person with ASD [3]. In light of this individual diversity, it is possible to find two children with the same diagnosis behaving completely differently from one another [4].

To determine a diagnosis on the ASD continuum, symptoms are required in two areas: (a) difficulties in communication and social interaction, and (b) repetitive behaviors and narrow interest areas, including sensory deficiencies [4].

The prevalence of ASD is constantly rising and is found today by the Centers for Disease Control [5] to be one in thirty-two children, suggesting a prevalence rate of 3.2% among children. ASD is more common in boys at a 4:1 rate [5] and is often accompanied by additional co-morbidities [6].

Due to scientific findings over the years, there is consensus on the importance and efficiency of early intervention programs for children with ASD [7]. Such interventions should be implemented within the child’s natural environments [8] and should be comprehensive by involving both their families and their direct and professional care providers, in accordance with the ecological approach [9]. Beyond choosing a specific therapeutic method, it is important to define clear therapeutic goals together with the family and to re-evaluate their implementation at regular intervals [4].

As reported by the Council on Children with Disabilities and Behavioral Pediatrics, evidence-based treatments for children with ASD can be presented as a Comprehensive Treatment Model (CTM) or as focused interventions. These treatments address the therapeutic, educational, and behavioral challenges presented by children with ASD. These interventions can be provided in different settings, by different providers, and can be implemented individually or in group settings. The most common reported intervention methods are Applied Behavior Analysis (ABA), Treatment and Education of Autistic and Related Communication-Handicapped Children (TEACCH), and the Early Start Denver Model (ESDM) [10].

### 1.1. Community Preschools Settings for Children with ASD in Israel

To enable these early and intensive interventions, Israeli children diagnosed with ASD are eligible for a diverse and significant therapeutic package, adapted to each child’s needs, of 14 h of health-related professional therapy weekly, financed by the Israeli Ministry of Health. They can have it either by integrating into a mainstream educational setting and receiving those therapies in the afternoon in the community health care clinics or by attending a segregated, communication-oriented special educational setting, where these children aged 3–6 years with ASD receive an intensive program by educational and health professionals [8,11].

Those intervention programs for children with ASD in the educational environment are tailored to their individual needs and include setting goals and selecting intervention approaches applied in the child’s natural environment [8]. These intervention efforts have been found to be effective [12]. Yet in practice, due to the growing number of children with ASD in need of a variety of treatments, there is a significant shortage of health professionals who can provide these intensive interventions, which fails mainly in the periphery. For example, in the year 2022, out of 6697 children diagnosed with ASD in Israel, over 800 of them did not receive services at all [12]. This major problem in providing adequate care for children with ASD can be partially solved by using telehealth services (for further information regarding the Israeli program, refer to references [12,13,14]).

### 1.2. Telehealth Service for Children with ASD

Different telehealth services have gained momentum, especially during the COVID-19 period, when most pediatric rehabilitation programs shifted to a virtual delivery format without the benefits of evidence to support this transition. As a result, it was found to be beneficial and effective in the treatment of children with ASD by many researchers [15,16,17] for children at different levels of ASD [18] while reducing costs and enhancing satisfaction [12]. These services, if properly implemented, can reduce the gap between the need to provide intensive care to children with ASD, a constantly growing population, and the lag in available professional care [19]. In accordance with the ecological approach, telehealth intervention may have a variety of advantages for the children themselves, their family members, and the educational staff. It offers the possibility of expanding and implementing an intensive therapeutic intervention in an ecological, all-day support program, in a cost-effective manner other than the traditional frontal interventions [2,3,4,5,6,7,8,9,10,11,12,13,14,15,16,17,18,19,20,21,22].

### 1.3. The Ecological Hybrid Intervention for Children with ASD

A new remote therapeutic service using internet technologies for children with ASD is called the Hybrid Ecological Intervention (HEI). The HEI model is both remote, more intense, and ecological. This approach is also more accessible for families and health care systems than the traditional individual, frontal treatment approach and is applied in a variety of situations and natural surroundings [19,23]. This model can be delivered either synchronously (implemented in real time) or asynchronously (implemented in a remote fashion by a clinician aid)**.** In the synchronic modality, video conferencing consists of face-to-face professional clinical meetings. In this modality of telehealth, client interactions occur in real time via a two-way video and audio interaction [19,24], and another adult (an in-person Educational Supporter (ES) is there to support the video recording of therapist\parent–child interactions.

The HEI method (which will be explained in full within the materials and methods section) uses both the benefits of face-to-face meetings and the qualities of the telehealth asynchronous approach (using existing, stored materials during face-to-face meetings).

### 1.4. Conclusions

There is a consensus that the treatment of a child with ASD should be intensive, implemented as early as possible, and within the child’s natural environment. In practice, there is a lack of health-related professionals who can provide in-person, frontal treatments for this constantly growing population, especially in peripheral areas. A possible answer to such a predicament is to activate a hybrid professional treatment model. The HEI combines remote intervention as well as bi-weekly individual frontal intervention, reinforced by an intensive ES, who performs daily exercises in the child’s natural educational environments, which is a new approach and is the focus of the current article. We hypothesize that by operating the HEI program, the model’s feasibility will be determined, the children will benefit from more intervention opportunities in their natural daily environments, and the new HEI approach will not be found to be more expensive than the conventional intervention method.

## 2. Materials and Methods

Ethics—The intervention was approved by the Ariel University IRB on 15 April 2023 (AU-HEA-ML-20230415) and by the Ministry of Education. The management of Tipuli-ad-habait company (TAHb) managing treatments within the participating kindergartens was also maintained. All parents of the children involved signed informed consent forms before initiating the intervention.

Aim—To evaluate the possibility of using a Hybrid Ecological Intervention (HEI) in the treatment of children with ASD to assess children’s improvements when using the HEI.

Research format—A baseline VS intervention format in education is a research design where baseline data is collected, followed by an intervention, thereby providing evidence of the intervention’s effectiveness when changes occur only after it is implemented [25]. This format was chosen due to the clinical heterogeneity of children with autism; the authors found it inappropriate to select a different group of children (presenting a completely different picture of ASD despite having the same umbrella diagnosis) to serve as a control. Therefore, a before-and-after study design was implemented. The decision to use a mixed-methods research design combining qualitative and quantitative data was made as this combination provides a more comprehensive understanding of a topic by offering a more holistic view. This approach can strengthen the overall rigor of the study. due to the novelty of the approach, enabling the program effectiveness to be shown while illuminating deeper qualities and challenges when implementing this approach [26].

Inclusion and exclusion criteria—The children within the participating kindergartens were all diagnosed with ASD, in accordance with the national diagnostic criteria (an ADOS evaluation, confirmed by an official diagnosis made by a child neurologist)**.**

The study population—The population consisted of children diagnosed with ASD according to standard diagnoses, integrated into communication kindergartens managed by TAHb in three different cities A (N = 8), N (N = 8), and R (N = 9), for a total of 25 children (see Table 1).

Study structure—The study was a combination of qualitative and quantitative elements using the Participatory Action Research (PAR) method, implementing a multiple baseline evaluation model. The qualitative part of the study included focus groups with the hybrid teams, for the purpose of building and following up on the GAS goals for each child and sample evaluation of videos.

### 2.1. Setting

“Tipuli-ad-habait” (TAHb) (name in Hebrew = treatments to your home) is a company devoted to treating children with ASD (https://tipul-li.com/). The company employs Speech Therapists (STs) and Occupational Therapists (OTs) within community clinics in combination with technological aids in communication centers, as well as developmental centers across Israel. The Goal of TAHb is to increase therapeutic interventions, thereby improving care quality indicators and professional training for the kindergarten staff, achieving a more continuous practice and a solid preschool framework as part of a complete ecological model that combines direct and indirect treatments in combination with the kindergarten staff and the child’s parents. The TAHb company operates 144 communication kindergartens and six early development therapeutic units across Israel.

### 2.2. The Ecological Hybrid Intervention (HEI) Model

The hybrid treatment in the framework of home care, which is intended to increase therapeutic interactions, improves quality indices of care and professional training for all kindergarten staff (education and health), achieves continuous and ongoing practice of treatment goals, and is presented to the kindergarten setting as a complete ecological model (direct and indirect treatment of the child and involving the kindergarten staff and parents).

**Figure 1:**
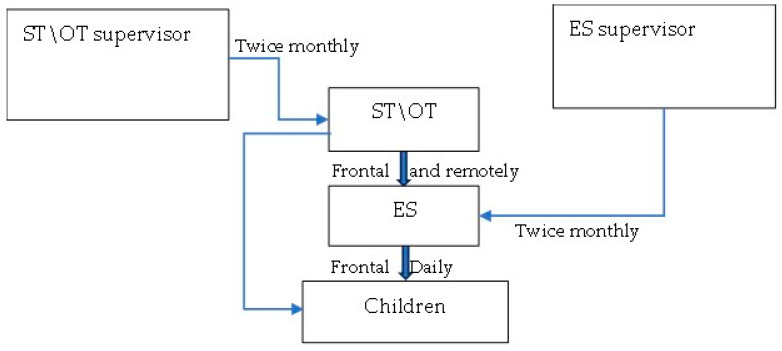
A flow chart describing the relations between the different position holders within the project.

The hybrid treatment was provided by a certified healthcare professional, a Speech Therapist (ST) or Occupational Therapist (OT) in cooperation with an Educational Supporter (ES) in accordance with the guidelines of the manual of the hybrid model treatments. The roles within the hybrid model and description of the role holders are presented in Table 2 and Figure 1):

(A) The clinical director serves as the research organizer and integrates and facilitates the therapeutic process. The clinical director’s position is not a usual position within the HEI structure and is only associated with the implementation of the research.
(B) The hybrid counselor is a field instructor with seniority and professional experience who will accompany the therapist and the assistant in the field, guide them, audit the process, and provide weekly feedback to the clinical director.
(C) The hybrid therapist is a certified health care practitioner (ST or OT) who will combine therapeutic sessions within a frontal framework (twice a month) and remote work (twice a week) with the help of the ES. The professional therapist guides the learning supporter to tailor an individual therapeutic program for each child.
(D) The educational supporter is a care provider who has undergone a short (one-day) instructional workshop on the technical operation of the different applications (Poppins). She accompanies the health care provider in her frontal and remote interventions and continues to work with the different children daily in accordance with the pre-determined individual program of every child.

### 2.3. Implementation Tools

Poppins (play and learn application)—The main system enabling this type of remote/hybrid intervention was a software application named Poppins. The Poppins app enables professional treatments in a variety of fields through a unique combination of an advanced digital platform. These treatments can be delivered at a time and place convenient for the patient and therapist with immediate availability. The system also follows up on the clinical progress of each client. The system is secure, meeting the strictest safety requirements and standards of the Israeli Ministry of Health. The Hebrew version of Poppins can be found at the following site: https://tipul-li.com/channels/poppins/ (accessed on 29 July 2025).

Currently, this technology is used by the four Israeli Health Maintenance Organizations (HMOs) and was implemented in accordance with the Israeli Ministry of Health guidelines on the subject.

Prior to the commencement of the therapeutic intervention, those involved received professional training in accordance with the Israeli Ministry of Health guidelines on remote care, which includes technical training for about an hour on the use of the remote treatment system (Poppins), professional training regarding the remote treatment protocol, and professional enrichment for all involved through academic courses dedicated to the field of remote therapeutic intervention with emphasis on ASD. In addition, the therapist and the technological assistant underwent individual training of one hour a week of professional guidance by an experienced therapist.

Cognishine learning program—Cognishine is an innovative digital intervention platform that empowers allied health professionals (e.g., speech and occupational therapists) to address the diverse needs of patients across various age groups, cultural contexts, and linguistic backgrounds. By digitalizing traditional therapy activities, Cognishine delivers an intuitive, customizable platform for both remote and in-person (one-to-one) therapy in different formations, such as one to many and group settings. Currently, Cognishine supports many languages, including English, German, Arabic, Russian, Hebrew, with expansions planned for Spanish, French, and Turkish. The app can be found at the following address: https://app.cognishine.com/en-us/ (accessed on 10 September 2025).

All participants had an hour of training in using the Cognishine platform before the initiation of the current intervention.

The authors had no contact or decision regarding the implementation tools (educational applications) and therefore no conflict of interest in choosing or using the above-mentioned tools.

### 2.4. Outcome Measures

Quantitative:

1. Communication and language skills—Each child’s functional level for language and communication was performed at the beginning of the academic year by using two scales:
  A. The Preschool Language Scales 5th Edition (PLS-5). PLS is one of the primary tools for identifying language disorders and determining eligibility for related services at ages 2–6/11 (years/months) [27]. It was approved for use by the Israeli Ministry of Health [28]. The PLS consists of two sub-scales—language comprehension and expressive communication. The PLS was translated and validated for use in Hebrew. When validating the PLS with participants diagnosed with ASD, it was recommended to use complementary language\communicative scales to complete the results of PLS [29]. Therefore, a second evaluation tool was used.
  B. The regular clinical evaluation form used in Tipuli’s communication kindergartens (not validated but used for several years with good clinical sensitivity and results) was used as an additional information source to establish communicative goals for each child. The communicational progress of each child according to his individual goals was evaluated monthly by the educational staff and validated through daily video recordings performed by the kindergarten staff during a free-play period at the beginning of each day.
2. Play skills—In the kindergarten where the focus of the research was on occupational therapist work, each child’s play skills were assessed at the beginning of the academic year by using two scales:
  A. Test of Playfulness—Play skills assessment was performed by using the Test of Playfulness Version 4.0 (ToP-4) [30]. The scale was used and validated for children with ASD [25]. The ToP-4 was translated and validated for use in Hebrew [28] and is recommended for use by the Israeli Ministry of Health [28]. As the TOP-4 mostly relies on verbal comments, a second evaluation tool was added to assist with individual quantitative analysis.
  B. A functional evaluation form used by occupational therapists in the kindergartens of the Therapeutic Association—The progress of each child according to his individual goals was evaluated monthly by the educational staff.
    The four above-mentioned scales are mostly verbal in nature, thereby complicating a quantitative result. In order to scale the GAS, in the current research, 90 s videos were filmed every morning at 08:30 a.m., during free play in the kindergarten, by the care-providing staff. In order to quantitively scale the PLS-5 and ToP-4, the same films were analyzed according to two individual goals (communication was measured by initiative/responsiveness, and playfulness was measured by the number of partners involved in the free-play session) constructed for each participating child by an objective reviewer (a speech therapist experienced in communication enhancement with children with ASD, yet not a person involved in the company that operated the kindergarten and in the research implementation). The above-mentioned videos were watched by the educational and therapeutic teams during the focus groups held during the research. The children in the videos were classified together until a point of agreement was reached.
3. Autism Spectrum Rating Scale (ASRS)—The current study used the abbreviated version (15 items) for preschoolers of the Autism Spectrum Rating Scale (ASRS). This tool enables parents and care staff to identify symptoms, behaviors, and related characteristics of children (ASD) and adolescents between 2 and 18 years of age. The ASRS [31] validity was established by moderate to strong correlations between the ASRS and other ASD-related measures, such as the Gilliam Autism Rating Scale Second Edition [32] and the Gilliam Asperger’s Disorder Scale [33]. Further validity evidence was provided in further research projects [31]. The tool was translated into Hebrew and underwent adaptation and validation processes and was approved for clinical use by the Ministry of Health [34]. In the current study, the scale was rated by the kindergarten teachers.

Goal Attainment Scale (GAS)—GAS is a well-known, established method of measuring individual progress against individual goals adapted to each child [35]. It is a mathematically based technique [36]. The psychometric properties of the scale were evaluated, and it was found to be reliable and valid [37], as well as responsive to change [36] while being used to assess a variety of topics with different populations across different settings [36,38]. GAS was reported as a promising measurement approach when assessing children with ASD [39]. GAS is a 5-point scale, with −2 representing the participant’s current functional level. A score of 0 represents the expected level of functional achievement at the end of the intervention program. A score of −1 is the score when achievements have been made from the baseline status, which did not progress to the full expected goal (0). If a patient achieves more than was expected, based on pre-established criteria, a score of +1 (more than expected) or +2 (much more than what was expected) is given, depending on the level of achievement. Available evidence supports GAS scaling reliability and validity [40]. When using the GAS in the current research, 90 s videos were filmed every morning at 08:30 a.m., during free play in the kindergarten, by the care-providing staff.

### 2.5. Qualitative Data

Regular Zoom meetings were held to support the implementation of the program. Focus meetings were implemented regularly with the Hebrides teams once every two weeks. From open-ended conversations, themes were extracted to assess the strengths and barriers of the HEI method.

Method: After receiving the written consent of all guardians, all children were evaluated before starting the intervention part using all assessment tools (see outcome measures), monitoring the implementation of the program on bi-weekly videos of the children throughout the period—the videos were analyzed according to GAS objectives built for each child and were re-evaluated at the end of the interventional part of the study (quantitative).

### 2.6. Preparation for the Research (Academic Year 2022–2023)

During this year, preliminary research procedures were carried out in one kindergarten. During this preliminary study, technical tests were carried out, such as the existence of a proper and stable Internet network for remote intervention management and the purchase of portable iPads. The use of iPads enabled ecological intervention across different settings within the educational facility. During this year, staff members were trained regarding autism and play, concepts of ecological treatment, guidance on collecting relevant information, use of evaluation forms required for research, and more.

### 2.7. Duration of the Study in 2023–2024 (10 Months)

In the first two months of the academic year (September–October), conversations with the parents, receipt of informed consent confirmations, initial observation of the child, and the first filling of ASRS by the treating team occurred; at the same time, personalized treatment plans and goals were constructed, including individual GAS goals in free-play videos inside the kindergarten in the morning. After two months, a second ASRS was filled out by the same care team to receive a baseline measurement for each of the children prior to intervention initiation. Then, the hybrid ecological intervention plan was implemented for six months, during which free-play videos inside the kindergarten were filmed each morning by the service providers and analyzed by an objective reviewer to assess improvement in play and communication. The quantitative section was collected through feedback from the focus groups of the teams, occurring every two weeks, including the research coordinator, STs, the OT, the ST and OT supervisors, and the Educational Support (ES) personnel. At the end of the intervention period, a third ASRS was delivered by the same teams, along with satisfaction questionnaires for parents.

### 2.8. Qualitative Procedure

Data collection

Focus groups were held from the initiation of the project and implemented twice monthly until the end of the academic year. The meetings included the research coordinator (recording and collecting the data), the therapists, the therapists’ supervisors, the ES, and the ES supervisor. During the meetings, an open discussion was conducted for data collection [41]. The open discussion enabled all participants to raise any topic they found appropriate and present their understanding of the situation [42,43]. This methodology aimed to create a safe environment where participants could discuss any topic with utmost openness [42,44,45].

Qualitative Data Analysis

Due to the novelty of the intervention procedure and since this new procedure changed the routine work model, the focus of the data analysis was on understanding all data related to the individual experience of each staff member, as well as the parents and children, with further interpretation and analysis by the researchers [46].

The data was analyzed using the six-phase inductive thematic analysis; a bottom-up approach to coding using inductive analysis and analysis driven by what emerges from the data [47,48] were themes and subthemes that were created. Becoming familiarized with the data by reading textual data (e.g., transcript of interviews, reading, and rereading ideas). The systematic data analysis began by generating initial codes. Recognizing shapes and shifting from codes to themes was carried out by the research assistant, who was following and organizing the intervention. Then the themes were reviewed, defined, and named, terminating the process by producing a report. To mitigate the potential for chance associations, a triangulation approach was employed, wherein the questions raised during the analysis were reflected during the ongoing focus group, and after minute corrections suggested by the participants of the focus group, all participants approved the end decision and consented on the suggested interpretation of the data [47,48].

### 2.9. Statistical Analysis

Calculations were carried out by SPSS 23.0 software. A criterion of α < 0.05 was set as the significance level for the results, for two missing data imputation procedures were applied. For testing normality distribution, a Kolmogorov–Smirnov Lilliefors Significance Correction was applied for the score variable. A linear regression was performed with repeated measurements by month. The confounders’ communication and play advancements, interactions that express the number of treatments each month were neutralized by including them in the regression to relate only to the effect of progress in the timeline. September and October were introduced as a control group with no intervention program and served as a reference for measuring progress in subsequent months.

## 3. Results

### 3.1. Quantitative Results

#### 3.1.1. Number of Therapeutic Sessions

When correlating the number of all treatments (See Table 3) with children’s improvement through individual improvement according to GAS results, we found a high and significant correlation (r = 0.86).

When correlating the number of treatments by the ES alone with children’s improvement through individual improvement according to GAS results, we also found a high and significant correlation (r = 0.81).

When correlating the number of treatments by the therapist alone with children’s improvement through individual improvement according to GAS results, we also found a very high and significant correlation (r = 0.95).

#### 3.1.2. Play and Communication

The PLS-5 and ToP-4 scales included a lot of verbal information, which made it difficult to translate to quantitative information. Therefore, the verbal reports of the PLS-5 and ToP-4 were transformed into numerical values (0–4) for each child by the therapists and kindergarten manager involved, in an open discussion entailing examples from the daily performance of the children (which was videoed daily to serve as a reference point). This procedure was carried out until an agreement was achieved, thereby transforming the verbal reports to mean numerical values (0–4) for each child. These procedures were used previously as reported within the Methods’ section.

A regression was performed with repeated measurements by month. The confounders’ communication and social interaction, and the amount of treatments each month, were neutralized by including them in the regression to relate only to the effect of progress on the timeline. August was omitted as the basis for measuring improvements. September and October were introduced as a control period with no intervention program and served as a reference for measuring progress in subsequent months. The regression coefficients show a significant improvement since October, when the intervention program was introduced, and have been on an upward trend since (see Table 4).

Regression with repeated measurements by month was performed. The results show that the power increases with the increase in exposure to the treatment plan. The confounders communication, namely interactions, which express the amount of treatments per month, were neutralized by including them in the regression to take only the effect of progress on the timeline. September and October were introduced as a control group without an intervention program and served as a reference to measure progress in subsequent months. The regression coefficients show a significant improvement since October, when the intervention program began to operate and have been on an upward trend since. The β improved in the last month (June) by 1.570 points compared to the control months of September and October. We should clarify that this is a linear GLM with repeated observations, and therefore, Exp(B) is not an odds ratio but rather a mathematical transformation of β and does not indicate multipliers of improvement.

**Chart 1:**
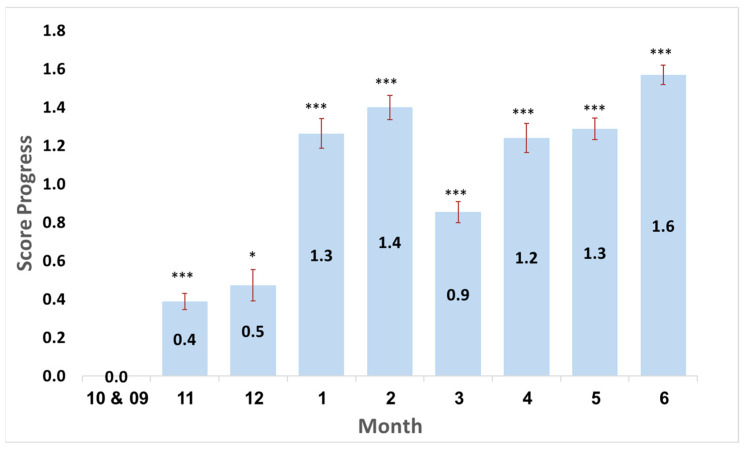
All kindergartens’ educational achievements mean monthly improvements. * < 0.05; *** < 0.001.

Chart 1, exploring monthly educational improvements through *β*, illustrates the increased cumulative improvement, given that all the months are significant compared to the control months used (September–October). These findings present the fact that the cumulative improvement index improved by 1.6 points in the last month of the academic year (June) when compared to the baseline period of September–October (no treatment).

#### 3.1.3. ASRS

The ASRS was implemented for children at all three kindergartens, suggesting

Repeated measurements regression was performed on the three ASRS measurements by month (Baseline, control—pre-intervention and post intervention). The confounder kindergarten was neutralized by including it in the regression. The baseline was used as a reference for measuring the group’s change in autism severity pre–post intervention (See Table 5).

The regression coefficients show a significant reduction in autism severity following the intervention program. The OR increased by 3.064-fold in the control group. However, this change is not statistically significant (*p* = 0.13), representing regular\common improvement achieved when children with ASD are enrolled in a communication kindergarten. In other words, without the implementation of the HEI therapeutic intervention, the improvement presented by the children was minute. In the control period (September–October), the score improved 11.2-fold when compared against the baseline/control period.

**Chart 2:**
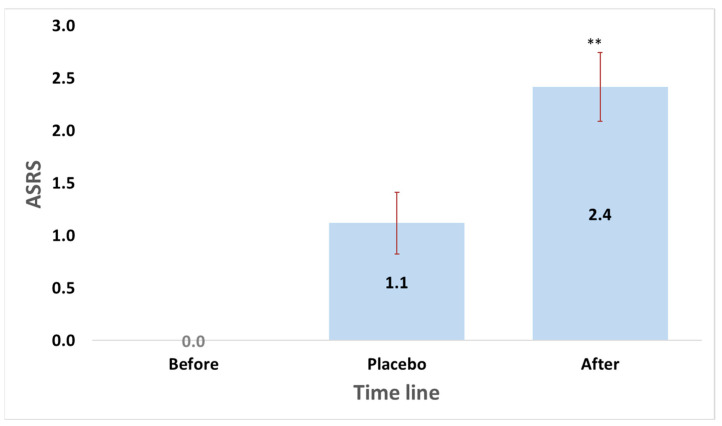
All children’s mean autism severity: baseline, pre-, and post-intervention, After ** < 0.01, Placebo < 0.05.

Chart 2 illustrates how the β ASRS score significantly increased post-intervention when compared to the change from baseline (pre-intervention). The score increased by 2.4-fold during the intervention period when compared to a 1.1 (non-significant) increase during the pre-intervention period.

#### 3.1.4. GAS

**Chart 3:**
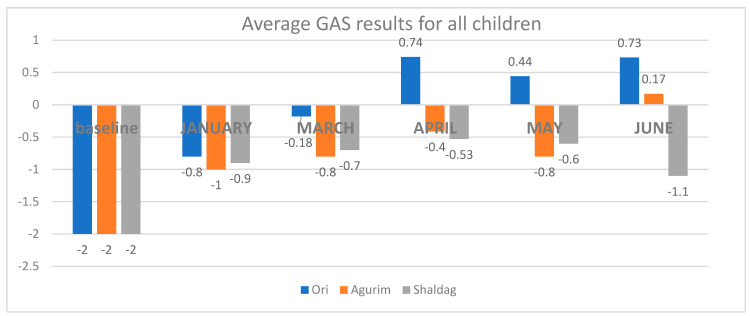
Average GAS results for all children.

The GAS results for all three kindergartens suggest a gradual improvement in individually adapted goals, especially in O and A kindergartens, with a decline observed in Sh kindergarten. The use of the GAS enabled showing no significant change pre-program initiation for both measured individual goals (*p* = 0.2; *p* = 0.1). This improvement trend was in accordance with the graphic representation, increasing gradually from baseline to June (*p* < 0.001), with a slight reduction in significance towards the end of the year. The change between baseline and June (end of the academic year) was still significant (*p* = 0.002; *p* = 0.001) (Chart 3).

### 3.2. Qualitative Results

The following themes emerged from the data analysis: connection between the health care professional and the educational support personnel, contact of the HEI team with the educational team, contact of the HEI team with parents, advantages of the HEI model, and challenges involved in implementing the HEI model.

#### 3.2.1. Connection Between the Health Care Professional and the Educational Support Personnel

All the participants involved discussed the necessity to formalize an ongoing positive relationship between the clinician and the ES from the beginning, in order to communicate with each other, even on days that are not defined as working days (24/7).

*A. (OT): “The essence of the model is based on a relationship of trust and perfect coordination with the ES.”*

*S. (ES): “We communicate a lot on WhatsApp and on the phone; when I have questions on non-working days, A. feels comfortable communicating with me. We don’t just call on Sunday-Thursday”.*

*A. (OT): We communicate all the time, with every question… If we were to maintain official boundaries, then the two half-hours of instruction are very necessary. We both feel comfortable communicating 24/7 as needed”.*

#### 3.2.2. Contact of the HEI Team with the Educational Team

The preliminary preparation of the educational staff for the HEI model was necessary since it is different from the conventional model with which the staff was familiar. The HEI team (the OT\ST\ES) implemented the model in the common shared space as opposed to the treatment rooms. This was possible when the relationship was good with both the HEI team and the educational team.

##### Flexibility Between Both Teams

The success of the model was based on the flexibility of both the HEI team and the education staff.

*Sh: “Yes, they understand the model, it doesn’t seem strange to them that I go out into the common shared Space with my iPad. Even during team meetings, A. can participate using the iPad, and her voice is heard”.*

According to A, “ Even though I only see them every two weeks, there is a good relationship [between us]. Each one gets the other’s professional place. At a staff meeting, even if I am at home, I will be asked my professional opinion… The relationship with the kindergarten staff works well. If a child has a difficult time going into the therapy session, the entire educational team helps. The kindergarten teacher asks me (A, the ES) for ideas and implementation of possible games and techniques. This is what I can name as ’Ecological’.”

She continues: “*When there is any difficulty with a child, they [the educational team] will volunteer to help me.” SH (OT Supervisor) A adds “Because there is excellent integrative work.*”

#### 3.2.3. Contact of the HEI Team with Parents

The parents were mostly satisfied with the HEI method, although the vast majority were not actively involved, by downloading the games and playing with their children at home.

##### Parental Satisfaction

Parents reported they were very pleased with the new model, yet most of them did not implement the HEI model at home.

S (ES) reported that “*During mid-term meetings attended by A. (OT) the parents were very pleased.” S also reported that the parents said “Most parents said: ’If I see progress, I’m calm’*.”

##### Parental Education

The model should be conveyed more clearly to the parents in continuous, regular contact, in order to enhance their active participation, thereby making the model completely hybrid (in the kindergarten but mostly at home).

*S: “The parents don’t know why I am there every day and why is A [the OT] only once every two weeks, but after A.’s mid-term meetings with the parents, they understood.*

*A.: “There is no regular weekly/daily contact [with the parents], they are updated once a month with a photo/video through the parent hub. Some parents want more while most want less. ”*

##### Individual Adaptation of the HEI Model to Each Parent

The relationship with the parents should be adjusted according to the parents (some prefer closer contact and some less).

*“A: [Usually] I send updates once in…to the parents. Occasionally, there is direct communication with a parent [involvement]…*

*Mo: [OT supervisor] recommended sending parents only a link to a game that was tried [online with the parents] to be practiced at home.”*

*A.: Some parents want more…The system enables the parents to watch the treatment with a closed camera for a few minutes.”*

#### 3.2.4. Advantages of the HEI Model

The HEI model intensified the number of treatments (some children receiving daily sessions) and enabled therapeutic meetings across different settings

##### Quality of the Intervention

Professional achievements were enhanced, and the sessions in the various kindergarten environments were found to be at a very high therapeutic level.

*S. (ES): A. [The OT] is really present. Thanks to the iPad in all kindergarten spaces, we are the champions of remote therapy.*

##### The Ecological Perspective of the HEI

The model can be operated throughout the kindergarten, including on the playground. This section of the kindergarten was mostly recommended by all the HEI model’s personnel as it assisted communication with low-functioning children who find it difficult to collaborate.

*S. (OT): In the playground, many people interact with U. (a low-functioning child), and it’s really significant because it’s hard to recruit him to pay attention in the therapy room, so in the playground, it was really successful.*

*S: “Children who do not want to enter the therapy room can manage to cooperate in other spaces. This is the essence of Ecological intervention”.*

*A (OT): Some children show difficulties in the therapy room, but they can be treated in the kindergarten’s common shared space.*

#### 3.2.5. Challenges Involved in Implementing the HEI Model

##### Technological Aids and Technological Issues

Implementing the HEI model necessitated constant, high-quality, reliable internet services.

*Sh. (ES): Usually, the internet connection works, but sometimes, it doesn’t work”*

*M (OT supervisor) states, “we overcame internet problems. The technological aids and the different educational Apps should be appropriate for the children at all ASD levels. She continues, “The games… are not suitable for low-functioning children. [These] games need to “open your mind” to know how to fit children at all functional levels.*

##### Difficulty for Other Children

Some children found it difficult to stay focused on their own tasks while other children were treated next to them within the kindergarten’s common shared space.

*S. (ES): “When A. (OT) is on mute and the camera [is] closed, it is possible to work in the open space. Otherwise, all the children are distracted by the iPad.”*

## 4. Discussion

The current research evaluated the feasibility and effectiveness of a novel method of intervention, the Hybrid Ecological Intervention (HEI) with children with ASD.

The results of both arms of the evaluation methods (qualitative and quantitative) suggested that the HEI method was found to be effective (when assessing the quantitative data) and feasible to integrate in a working framework (by assessing the qualitative data) in this group of clients.

### 4.1. Quantitative Data

#### 4.1.1. Evaluation Methods

The following scales were used within the current research’s protocol: the Preschool Language Scales 5th Edition (PLS-5), the Test of Playfulness Version 4.0 (ToP-4), the Autism Spectrum Rating Scale (ASRS), and the Goal Attainment Scale (GAS).

The PLS-5 and ToP-4: Both scales include a lot of verbal information, which made it difficult to translate to quantitative information, as explained within the results section. The findings in the current research suggest that both scales are meaningful more as individual tracking of each child when compared to himself rather than as a group evaluation within a research context (see Chart 1). Similar procedures were used by others when extracting individual results into group findings when using the PLS-5 [49] and ToP-4 [50,51]

ASRS: The ASRS (see Table 1 and Chart 2) was found to be extremely accurate in measuring the results of the current study. This is not a surprise, as this scale is widely used in autism research [31,32,33,34] and is therefore highly recommended for future use when performing interventions in children with ASD.

GAS: This scale that has existed for the past 50 years and was found very useful in the current study (Chart 3) as well as by others [35,36,37,38,39,40]. The ability of GAS to be used to assess improvement in any field you choose is extremely adaptable to the area of autism, where heterogeneity between children is so obvious and therefore necessitates a scale that can collect an assortment of goals (different and unique to each participant), and at the same time, can collect all the data into one group of results. The authors highly recommend the use of GAS in interventional research projects in the future, especially when being applied to heterogeneous groups of clients, such as those with ASD.

#### 4.1.2. Improvement of the Participants Involved

All the quantitative evaluation methods were implemented before (baseline) and during the implementation of the HEI model. All the above-mentioned scales presented improvement of the participants involved regarding their play and communication skills (assessed by using PLS-5 and ToP-4), their autism severity (assessed by using ASRS) and individual development (assessed by using GAS). These findings express the difference between achievements in the above-mentioned areas prior to the implementation of the HEI model (not significant) and therefore support the use of the HEI model in kindergartens of children with ASD. Yet these findings are enriched and give significant insights into the benefits and challenges of the HEI through the focus groups held by the HEI and educational teams.

### 4.2. Qualitative Data

The following section will discuss the findings of the qualitative data and their clinical implications.

#### 4.2.1. The Connection Between the Health Care Professional and Educational Support Personnel

Within the HEI, formalizing a good rapport between the clinician (OT\ST) and the ES from the beginning of the project was found to be necessary. This is necessary in order for communication to occur between each other even on days that are not defined as working days (24/7). Similar results were found in research assessing the difference between face-to-face intervention for children with ASD vs. remote intervention [52], where participants reported that remote intervention enhanced the connections between team members. This finding was true for the current research, where parents were invited to see their children during therapy sessions and staff members were able to participate in staff meetings without leaving their homes.

#### 4.2.2. Contact of the HEI Team with the Educational Team

There is a need for preliminary preparation of the educational staff for the hybrid model because it is different from that with which the staff is familiar. In the HEI model, the OT\ST\ES implement the therapeutic sessions in the common shared space as opposed to the treatment rooms. Therefore, when the relationship is good, the entire team is recruited. Moreover, due to the importance of this issue, a preliminary pilot was held in the year preceding the reported project, as recommended by the Israeli Ministry of Health [9] when performing remote rehabilitation interventions with children with ASD. Such recommendations should be intensified in the case of HEI, as this complex model incorporates two different working models (remote and face-to-face), working in different settings (in the therapy room as well as open spaces within the kindergarten) and incorporates parental involvement during therapy sessions.

Such a process is also suggested by others since a collaborative approach between professionals in an educational center leads to improved client and student outcomes [53]. Further readings regarding actual techniques for enhancing inter-staff collaboration can be found in Hart and O’Shaughnessy [54].

#### 4.2.3. Contact of the HEI Team with Parents

In general, parents were very pleased with the model. Yet, in the current intervention period, most of them refrained from using all the benefits of the HEI. The full HEI model enables parents to join therapeutic sessions while the child is at the kindergarten and appreciate his interests and abilities as well as to become a part of the child’s ecological experience. The parents could also intensify the treatment and their connection by using the educational application at home with their children as a recreational activity. In order to achieve the maximal benefits of the HEI, more regular, intensified contact is necessary between parents and HEI staff. Intensified parental education is also suggested by the Israeli Ministry of Health when working with children with ASD [9]. Improved recruitment of parents into the ongoing intervention procedure, parent guidance regarding the importance of such an involvement, and technical support available to parents are needed in future projects to enhance the project’s capabilities and ecological values.

#### 4.2.4. Advantages of the HEI Model

When conducting the interviews with the HEI staff, the following benefits were found:

- **Intensifying intervention**—The model intensifies the intervention, exposing the children to daily promotion of educational, fun exercises. Intensifying intervention with children with ASD is a common understanding accepted among all care providers working with this group of clients [7,11,55].
- **Functional achievement**—The quantitative methods we implemented during the current research suggest that significant achievements were made by the children. The sessions in various kindergarten environments attest to the model’s feasibility and contribution within kindergartens of children with ASD.
- **Ecological implementation**—The model can be operated throughout the kindergarten and specifically within the playground. This section of the kindergarten was recommended by participating HEI personnel in all kindergartens. The use of the ecological approach is also recommended by others [56], thereby supporting the use of an ecological model with this group of clients.
- **Application with low-functioning children with ASD**—The interviewees of the current project attest to the fact that operating the ecological system in a more natural environment (such as the playground) might assist communication with low-functioning children who find it difficult to collaborate when the therapeutic intervention is applied in the therapy room [57].
- **Technical solutions**—The implementation of such a unique novel intervention requires good educational applications and constant technical support in order to enable remote access for the HEI team.
  Using tablet devices (rather than stationary computers) made it perfect for changing the site of the interaction with the different children, thereby making it truly ecological.
- **Good working relationships**—Good working relationships between the educational team and the HEI team were found to be essential for the success of such a program.

#### 4.2.5. Challenges Involved in Implementing the HEI Model

- **The need for appropriate educational applications**—Some of the educational programs used in the current research (Poppins and Cognishine) were found by the users not to be completely adapted for children in kindergartens, especially for low-functioning children. Therefore, there is a critical need to further development of newer, more appropriate educational applications suitable for children with ASD at all levels of social and cognitive abilities. Such programs should include activities addressing many areas of interest, thereby promoting a high level of motivation. Similar suggestions have been raised by others [58].
- **The need for highly trained personnel**—When applying the HEI, all the involved personnel have to be flexible. This type of flexibility necessitates high-quality professionals, together with appropriate pre-intervention training in order to achieve maximal results. The need for highly trained personnel when working with these children has also been echoed by others [59]. Moreover, the staff must also be technologically savvy in order to use the applications, as well as to solve technological challenges when these arise.
- **Disturbing other children**—When implementing the model in all kindergarten surroundings, other children who are not the direct recipients of the therapeutic intervention might be distracted by the iPad. This can be avoided through technical solutions (by operating the iPad in mute mode and with the camera closed).
- **Technical difficulties**—The implementation of the HEI requires a constant, reliable internet connection. Therefore, the use of such technologies in developing countries might be problematic without a robust, constant internet infrastructure. Such concerns have been raised in the past [60].

### 4.3. Limitations of the Current Research

- The small number of participating children—As this was a feasibility study, the number of children was small, with the intention of expanding this format of intervention to more kindergartens in the future in case of favorable results, which were achieved.
- Performing the program during war times—the implementation period (wartime) could not have been anticipated and has surely prevented even better results related to the HEI program, yet the favorable results achieved in an unstable period further strengthen the findings and support this form of intervention.
- Full ecological implementation—The HEI method was intended to also include parental involvement at home. Yet, the participating parents were reluctant to take part in the home-based part of the procedure. Despite the lack of parental involvement, the results support the power of the HEI method. When implementing the HEI model in the future, efforts should be made to enhance parental involvement. Parental involvement steps should include improved recruitment procedures, understanding the importance of their involvement, and suggesting technical support.
- Lack of a control group—Due to the clinical diversity presented by most children with ASD, a comparison\control group was deemed inappropriate by the authors. Therefore, the use of a baseline period followed by an intervention was applied to assess participants’ improvement under the current intervention protocol with the same children before the intervention implementation (baseline period) and during the implementation of the intervention protocol.
- Single-country context—As this is a feasibility research, it was conducted in just a few kindergartens in one country, and therefore generalization is limited. Future evaluation of the current method should be implemented in a multiple-site method, assessing its feasibility in different frameworks and situations.

## 5. Summation

The results of both parts of the current research suggest that the Hybrid Ecological Intervention (EHI) is feasible in kindergartens of children with ASD. In the HEI model, children received a high number of therapeutic meetings (of different sorts) and enhanced intervention. Despite its intensity, the costs of the current program (HEI) were kept at the same cost level as a regular model, wherein children receive fewer treatments weekly.

Future interventions should apply a pre-intervention period to resolve all possible challenges (technical and personal), and there should be a multi-site (country) implementation to assess feasibility in different frameworks and mentalities that includes more children to enable generalization and rigorous support of the findings and to provide more support to the parents so that the project can become truly ecological.

## Figures and Tables

**Table 1:** Demographic data for participating children.

Kindergarten Name	# of Children	City Socioeconomic Scale	Mean Age (Months)	Age Range (Months)	Females	Males	Sector
A	8	5	70.1	63–76	0	8	ST
Sh	8	6	44.3	39–52	0	8	ST
O	9	6	44.8	40–51	2	7	OT
Total	25	---	53.1	39–76	2	23	

Index—#—Number. City socioeconomic scale—The socioeconomic scale is set between 1 and 19, with 1 being the lowest possible score, suggesting that the three participating kindergartens are in cities within the third-lowest economic status in the country.

**Table 2:** Demographic data for participating clinicians.

Kindergarten Initials	Initials of Care Provider	Profession	Duration in Profession (in Years)	Duration of Work with ASD (in Years)	# of Meetings in a Month
A	RM	ST	3	3	16
	OE	ST Supervisor	20	15	Once weekly
	LB	ES	10	2	16
	SC	ES Supervisor	10	10	Once weekly
Sh	MP	ST	5	2	16
	OE	ST Supervisor	20	15	Once weekly
	NDV	ES	---	1	16
	SC	ES Supervisor	10	10	Once weekly
O	SBD	OT	3Y	3	16
	MZ	OT Supervisor	9	7	Once weekly
	SA	ES	---	1	16
	SC	ES Supervisor	10	10	Once weekly
Mean					
Index: ST = speech therapy		ES = educational support		OT = occupational therapist	

**Table 3:** Number of therapeutic sessions.

Initials of Kindergartens	Total # of Therapeutic Sessions	Total # of Therapeutic Sessions by Therapist	Total # of Therapeutic Sessions by ES	Mean # of Monthly Therapeutic Sessions	Mean # of Monthly Therapeutic Sessions by Therapist	Mean # of Monthly Therapeutic Sessions by ES
**A.**	420	275	145	26.3	34.4	18.1
**S.**	338	233	105	21.1	29.1	13.1
**O.**	736	303	433	46	37.9	54.1
**Mean**	498	270	228	31.3	33.8	28.4

Index—# = Number.

**Table 4:** Education improvement within the intervention period.

Dependent Variable	Score Type	Parameter	*β*	95% Wald Confidence Interval		Hypothesis Test	Exp(B) = OR	95% Wald Confidence Interval for Exp(B)	
				Lower	Upper	Wald Chi-Square Sig.		Lower	Upper
**Manual Dexterity Eval**	Standard	Intercept	1.363	1.152	1.575	0.000	3.909	3.164	4.829
		Study Group	0.325	0.047	0.602	0.022	1.384	1.049	1.826
		Control Group	0.067	−0.227	0.361	0.653	1.070	0.797	1.435
	Percentile	Intercept	1.533	1.324	1.742	0.000	4.632	3.758	5.708
		Study Group	1.269	1.032	1.505	0.000	3.557	2.808	4.506
		Control Group	0.277	−0.003	0.558	0.053	1.319	0.997	1.746
**Aiming and Catching Eval**	Standard	Intercept	1.705	1.527	1.883	0.000	5.500	4.602	6.573
		Study Group	−0.008	−0.261	0.244	0.949	0.992	0.770	1.277
		Control Group	0.087	−0.160	0.334	0.489	1.091	0.852	1.396
	Percentile	Intercept	2.561	2.439	2.683	0.000	12.950	11.465	14.627
		Study Group	0.189	0.018	0.360	0.030	1.208	1.018	1.434
		Control Group	0.091	−0.075	0.258	0.282	1.096	0.928	1.294
**Balance Eval**	Standard	Intercept	0.841	0.566	1.115	0.000	2.318	1.762	3.050
		Study Group	0.741	0.408	1.075	0.000	2.098	1.503	2.929
		Control Group	0.243	−0.124	0.609	0.195	1.275	0.883	1.839
	Percentile	Intercept	1.476	1.145	1.807	0.000	4.375	3.141	6.093
		Study Group	1.418	1.066	1.770	0.000	4.130	2.904	5.872
		Control Group	−0.036	−0.495	0.424	0.879	0.965	0.610	1.528
**Total Test Score Eval**	Standard	Intercept	0.916	0.652	1.181	0.000	2.500	1.919	3.256
		Study Group	0.481	0.145	0.817	0.005	1.618	1.156	2.265
		Control Group	0.136	−0.226	0.497	0.462	1.145	0.798	1.645
	Percentile	Intercept	0.742	0.314	1.170	0.001	2.100	1.369	3.221
		Study Group	1.587	1.131	2.043	0.000	4.889	3.099	7.713
		Control Group	−0.049	−0.615	0.517	0.866	0.952	0.541	1.677

**Table 5:** ASRS changes during the intervention compared to the baseline period.

Dependent Variables	*β*	95% Confidence Interval		Hypothesis Test			Exp(B) = OR	95% Wald Confidence Interval for Exp(B)		Observed Power
		Lower	Upper	Wald Chi-Square	d. f.	Sig.		Lower	Upper	
(Intercept)	29.747	29.590	29.904	1915.083	1	0.000	*	*	*	1.000
Study Group	2.417	2.087	2.746	8.999	1	0.003	11.208	2.311	54.361	0.872
Control	1.120	0.825	1.415	2.306	1	0.129	3.064	0.722	13.003	0.389
Base line	0									
Kindergarten 3	−1.741	−1.997	−1.486	8.513	1	0.004	0.175	0.054	0.565	0.611
Kindergarten 2 1n	−1.843	−2.175	−1.510	5.136	1	0.023	0.158	0.032	0.779	0.052
Kindergarten 1 1n	0						1			

* An extreme number.

## Data Availability

Data available on request due to restrictions.
